# Evaluation of filler activation for sustainable FRP composite by studying properties, mechanism, and stability

**DOI:** 10.1038/s41598-024-68251-8

**Published:** 2024-08-06

**Authors:** Hamdy M. Naguib

**Affiliations:** Department of Petroleum Applications, Egyptian Petroleum Research Institute (EPRI), Nasr City, 11727 Cairo Egypt

**Keywords:** Fiber glass, Biological stability, Mechanical testing, Activation, Chemistry, Materials science

## Abstract

The aim is to develop new fiber-reinforced polymer (FRP) water pipe by activating fiber glass (FG) by vinyltriethoxysilane (VS) getting vinylsilane-activated FG (AFG) for filling vinylester (VE) via continuous winding to make a novel VE-AFG composite. The novelty of this work is the activation of fiber glass by vinylsilane as a single filler in vinylester and compounding them via a two-dimensional continuous winding process for the first time. The crosslinking occurred in the AFG/VE/curing agent system after activation. The activated composites increased thermal stability; 25% VE-AGF increased the degradation temperatures at 10%, 25%, and 50% weight loss by 73.3%, 10%, and 7.2%. With the activated 20% composite, values of axial strength, hoop strength, and hardness were developed by 6.3%, 2%, and 8.7%, respectively. The decay resistance to different microorganisms was increased with VE-AFG composites as a result of a sharp decrease in biodegradability percentages. The activated composites are stable toward water absorption; the least percentage was recorded by 25% VE-AFG, which minimized the water absorptivity by more than 62%. The reported characterization sentence approves enhancement of thermal, physical, and mechanical stability of sustainable vinylester-fiber glass composites manufactured by continuous winding; this is recommended for application in water pipe systems.

## Introduction

In recent decades, fiber-reinforced polymer (FRP) composites have gained great interest due to the variety of their applications^1^. In such FRP composites, the polymeric matrix may be thermosetting or thermoplastic polymers. The fiber filler may be fiber glass, carbon fiber, aramid, natural fiber, polyester fiber, polyethylene terephthalate fiber, basalt, etc.^2–7^. The preparation and development of related composites are currently reported. Fiber glass (or glass fiber)-based FRP is considered the most applicable composite due to its availability, low cost, low density, processing facility, chemical resistance, fire resistance and durability. Mainly, the fiber glass consists of melted silica-alumina admixture in different shapes^8–11^. Furthermore, the superior properties of vinylester matrix, such as surface stability, transparency, and brittleness, could enrich its applications. Paints, civil infrastructure, spacecraft, automotive, water pipes, laminates, wind turbine blades, etc. are known applications^12–18^. Some fibrous composites were developed recently for improving the performance of recycled reactants; the obtained products can be applied as useful recycled sustainable composites. Fiber glass is usually wetted with polyester/epoxy bipolymer matrices for the evaluation of composite^19–22^.

Related literature focused on the development and promotion of FRP. Hybridized glass fiber/screw pine fiber was used for filling vinylester resin throughout hydraulic compression processing. The dual filling has increased the level of shear property, especially with the skin–core type composites, compared with that of dispersed type composites^23^. In a new composite, the effect of the styrene/methacrylic acid diluent on the properties of vinylester-glass fiber composites was studied. Changing in glass transition temperature and mechanical behavior were reported; also, the diluted polymers showed good fire-retardancy due to the better wettability of fiberglass by vinylester^24^. A study focused on evaluating and simulating the effect of CNT on the performance of E glass fiber-reinforced polypropylene FRP. The treatment with functionalized multi-walled CNT was found to increase the durability of composites, which serve as improved wind turbine blades^25^. A glass/basalt fiber-reinforced vinylester/epoxy composite was formulated, and immersed in a seawater environment for different times to test the physical and mechanical degradation. With increasing aging duration, a steady decrease in tensile, flexural, and impact strengths with seawater aged laminates was noticed^26^. The durability of unidirectional glass fiber-reinforced polypropylene composite was checked under seawater sea sand concrete; it was found to be susceptible to high temperature immersion with better mechanical retention. However, it resisted corrosion poorly due to the bad attachment between glass fiber and matrix^27^. A new type of liquid DOPO containing vinylimidazole salt was successfully prepared and applied to flame-retardant vinylester-glass fiber composites^28^. For more advanced applications of FRP, different glass-based polymers and their nanocomposites were applied as crashworthiness materials in devices, vehicles, and pipes due to their improved energy-absorbing and quasi-static crush response properties^29,30^.

**Scheme 1:**
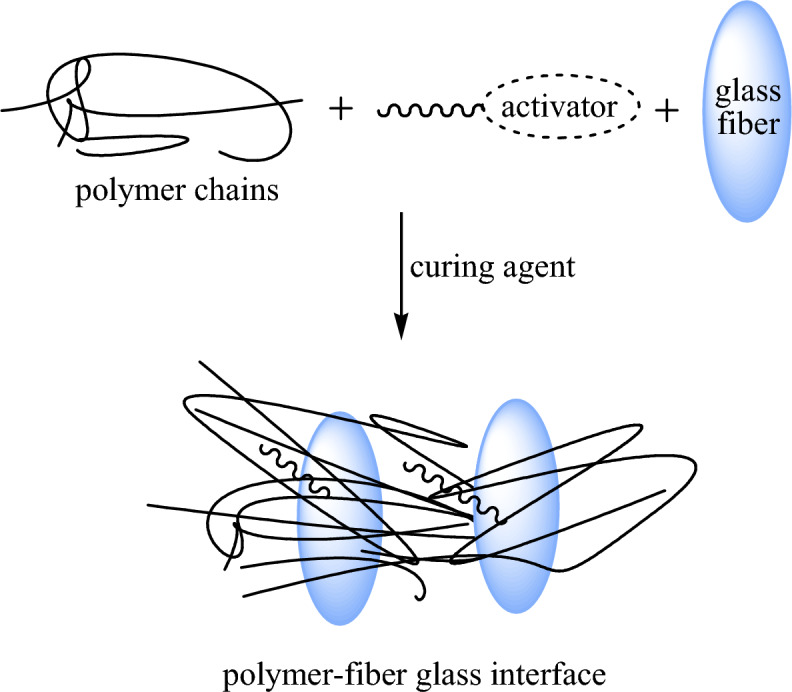
Fabrication reaction of vinylester polymer with activated fiber glass to form the vinylester-fiber glass composite.

Taking into consideration the possible combination between polymer matrices and used fibrous fillers as organic–inorganic hybrid composites, numerous ways of treatments and activations, may be chemically, physically, or mechanically, are executed on the reactants for a better filling effect. Of them, hydrolysis, irradiation, coupling, etching, grinding, heating, and cold plasma are taken as activation/treatment methods^31–35^. The obtained composites gather enhanced characteristics due to activations compared with unactivated ones. In other cases, second additives were loaded in the matrix rather than the treatment process to achieve the desired properties. In the same way, the effect of adding calcium carbonate and alumina trihydrate secondary fillers for making glass fiber-reinforced polymer gutters was found to increase density, hardness, strength, and elasticity, and to decrease water absorption^36,37^. However, this may generate some difficulties during processing due to the differentiation in their nature. Techniques of hydraulic compression and hand-layup are well-known for preparing different formulations of fiber-based vinylester composites^38^. Due to insufficient information about formulating vinylester-fiber glass composites via the continuous winding technique, water pipe specimens are prepared in the present work. A description of the proposed fabrication reaction is provided in Scheme 1. Fiber glass is activated and filled with vinylester polymer chains, and then the composite is cured as a hard vinylester-fiber glass composite interface. The effects of activation on the physical, mechanical, and biological properties of the obtained composites are examined.

## Experimental

### Materials and methodology

The chemicals and methodological process of the proposed composites are indicated: SWANCOR 911-grade bisphenol-A-epoxy vinylester (VE) is the polymer matrix with a viscosity of 550 cPs, a flash point of 35 °C and a styrene monomer of 40%. China Beihai fiber glass (FG) threads are the filler, with a density 2.55 g/cm^3^ and softening temperature 850 °C. Actually, the grades mentioned were selected because they are recommended for application with the glass fiber. This grade is compatible and sticky with fiber, so the fiber glass filler can absorb the resin completely. In the proposed work in the manuscript, the resin penetrated the fiber, leading to an enhanced and uniform composite, especially after the recommended activation reaction, as indicated later. Akzo Nobel methyl ethyl ketone peroxide is the catalytic agent. Hengda vinyltriethoxysilane was the activator compound used for fiber glass filler. Acetic acid was used for hydrolysis, purchased from Qingdao Hisea Chem Co., Ltd. All chemicals and materials were applied as received, except for the portion of fiber glass that underwent an activation reaction. For methodology, the activation of FG was performed first as follows according to reports elsewhere^39,40^. The activation solution, with a concentration of 0.5%, was prepared by stirring VS with a methanol-deionized water mixture (95:5%). The homogenous solution was then hydrolyzed at room temperature for 80 min by controlling the pH at 4. This step is to control the product as activated silane. The desired amount of FG was then soaked in the activation silane solution with gentle stirring for 30 min; the treated filler was finally dried, resulting in activated fiber glass (AFG). The VE-FG and VE-AFG composites with concentrations of 20% and 25% are selected for a full study on the effect of activation reactions. The composites were processed using the technique of continuous winding. A well-mixed matrix solution, prepared from vinylester resin and peroxide catalyst (100:2%), was poured on the stainless steel mandrel loaded with fiber glass fixed in axial and hoop directions. The obtained composite pipes were taken as the analysis standards after 15 min of working, and curing at 50 °C, then at room temperature for 24 h. The effectiveness of the activation reaction on the physical, mechanical, and biological activity of the proposed composites, in comparison with blank polymer, is debated hereinafter.

### Characterizations

The following analysis techniques were used for characterizations: for thermal stability, the Mettler Toledo SF-TGA analyzer was used by heating samples to 600 °C with a 5 °C/min rate in nitrogen chamber. For mechanical and surface properties, XLC H-universal testing machine, the MR SD-101 Split disk machine, and the Gardco-GYZJ 934 Barcol impressor have been used for testing the axial tensile strength, hoop tensile strength, and surface hardness, respectively, following ASTM D-638, ASTM D-2290 and ASTM D-2583 standards. The biodegradability testing was performed by immersing composites in different microorganisms’ media. Different bacterial strains were obtained as *Bacillus subtilis*, *Escherichia coli*, and *Candida albicans* and grown in nutrient broth medium for maintenance and cultivation at 30 °C for 30 days; the percentage of biodegradability was investigated by the mass change method using Eq. (1)^41,42^, where W_dry_ and W_bio_ respectively denote the weight loss of unconditioned and biological-conditioned composites. The stability of prepared composites toward water was checked by measuring their water absorptivity percentages (WA%) using Eq. (2), where wet wt. and dry wt. represent, respectively, the composites’ weights after and before wetting in the distilled water.

1 $$Biodegradability \%=\frac{{W}_{dry}-{W}_{bio}}{{W}_{bio}} \%$$

2 $$WA\%=\frac{wet wt. - dry wt.}{dry wt.} \%$$

## Results and discussion

### Curing reaction

**Figure 1:**
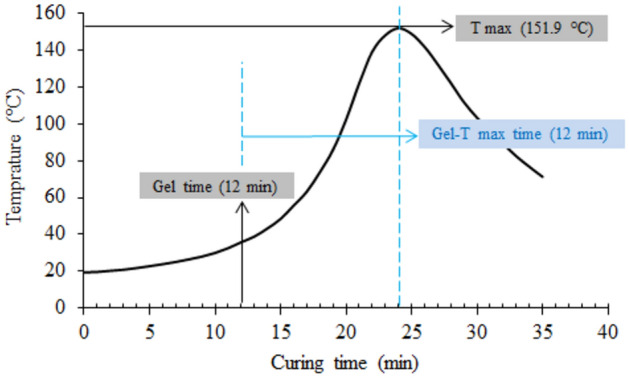
Thermo-physical properties of vinylester during the curing.

Upon curing of vinylester matrix, the reaction temperature gradually increases from room temperature to a maximum degree “T max” during the in-situ polymerization reaction after adding the catalyst. Also, the viscosity of vinylester matrix simultaneously increases, reaching the gel-like phase. The time taken to the later phase is called “gel time”, and the time taken from gel phase to T max is named “gel-T max time”^43–46^. As the main aim is to study the effect of fiber glass and its treatment on the mechanical and physical properties of the final composite, the concerned measurements during curing (after addition of curing agent) are studied here, regarding “gel time”, “gel-T max time”, and “T max”, to facilitate the addition and distribution of fiber filler before reaching the gel phase. These thermo-physical values characterize the processability of the matrix used, where the fiber glass fillers should be incorporated before the gel time. This assures a complete distribution inside polymer; the final composite has its ultimate properties as well. The overall properties of the cured composite are related to the concentrations of silane activator, styrene monomer, and catalyst, along with the viscosity of vinylester matrix. The mentioned thermo-physical properties are illustrated in Fig. 1, which shows the reactivity curve of vinylester. It is clear that after reaching the T max, 151.9 °C, within the time taken for curing due to the exothermic crosslinking, the system temperature was decreased dramatically to room temperature. 12 min was recorded for both gel and gel-T max times.

**Scheme 2:**
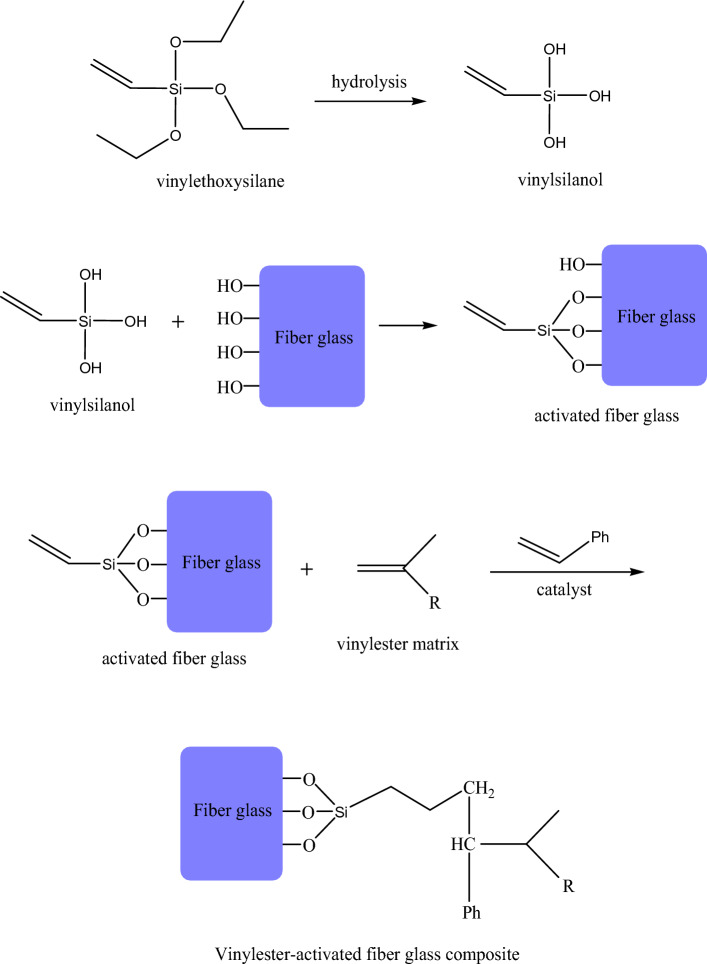
Proposed scheme for the interaction among vinylester matrix, fiber glass filler and vinylsilane activator.

For the activated composites, the proposed treatment and crosslinking are explained as follows: firstly, the vinyltriethoxysilane material undergoes a hydrolysis reaction to get vinylsilanol derivative, which is able to react with the fiber glass via hydroxyl functional groups through a condensation reaction. This step could motivate the surface of fiber glass, which is activated and functionalized by vinyl and hydroxyl active groups, resulting in activated fiber glass. In the case of filling vinylester with the obtained activated filler, the free radicals start to attack the unsaturation sites in vinylester matrix and activated fiber glass concurrently, in addition to the styrene monomer component. The final product is a crosslinked vinylester-fiber glass composite; this mechanism is represented in Scheme 2. The obtained vinylsilane-activated fiber glass can attach to the polymer matrix with more compatibility. In other words, hydrolysis and activation can facilitate the interaction between inorganic filler and organic matrix for improved composites. These treatments support the used fiber glass filler with more active moieties, which cause effective filling within the vinylester matrix; the cured network composite system has outstanding characteristics as well. During crosslinking, the addition of curing agent increases the viscosity as the crosslinking density increases until the gel-phase. In fact, the crosslinking density is the essential parameter that controls gel formation as precipitated molecules in the form of 3-D ordering^47^. The curing is considered an intermolecular reaction that leads to the formation of branched 3-D chains and larger network molecules containing polymer, curing agent and fiber glass filler, as provided in Scheme 2.

### Thermal properties

**Figure 2:**
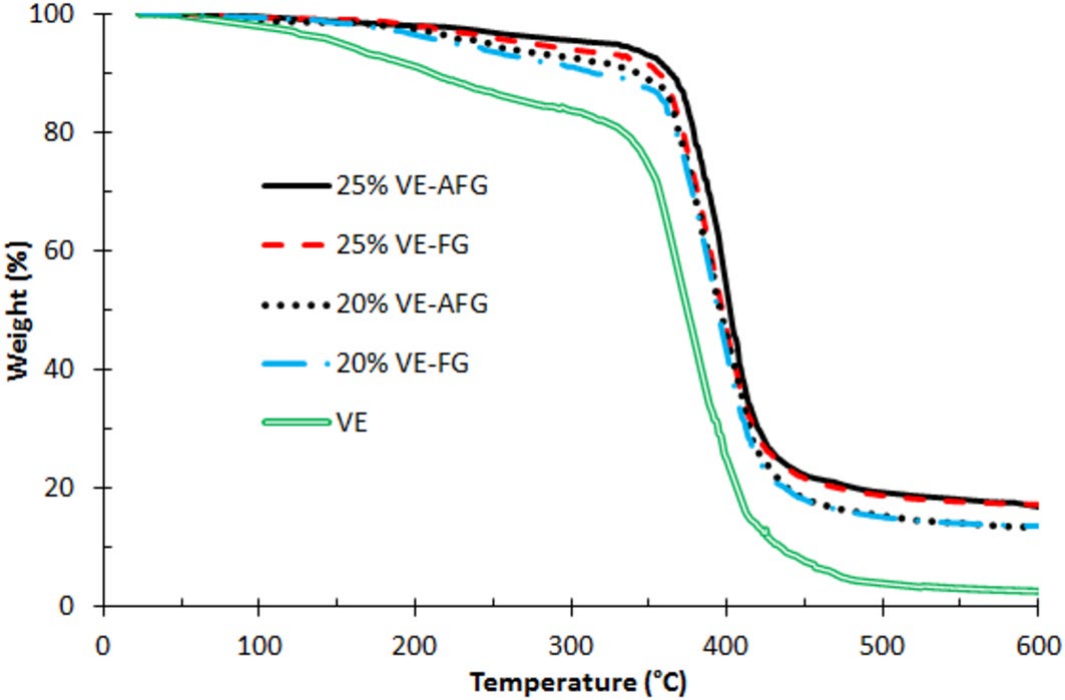
TGA of VE-FG and VE-AFG composites.

In Fig. 2, indicating the thermal gravimetric analysis (TGA), the effect of activating GF by VS on the composite is shown. Firstly, all composites show improved thermal properties in all ranges of temperatures compared with blank VE matrix. This results from the successful filling processing technique in all composites, in spite of the activation reaction. The glass fibers usually enhance the thermal profile of different polymer matrices^48^. Regarding the selected concentrations of composites, 20% VE-AGF only has somewhat higher degradation temperatures than 20% VE-GF within the range of 250 °C and 360 °C. This little increase is owing to the activation reaction. The effect of activation on thermal stability is notable in the 25% composite, where it shows higher degradation temperatures at larger range of temperatures, i.e., 240 °C and 400 °C. The reaction between vinyltriethoxysilane and glass fiber has strengthened the composite interface, leading to deformation at higher degrees. It was reported that the surface treatments of filler have delayed degradation and cleavage of polymer segments^49,50^. The thermal improvement in the latter composite may be due to the sufficient fiber content that is able to reinforce vinylester matrix, besides the activation reaction.

For detailed discussion, the primary degradation temperatures and residual percentages of the VE-FG and VE-AFG composites are presented in Table 1. The degradation temperatures at 10%, 25%, and 50% weight loss and the residual percentage are coded with T 0.1, T 0.25, T 0.5, and Res%, respectively. The degradation temperatures were increased after activation with VS in both composites. A large difference was realized by the 25% VE-AGF composite, where the mentioned values increased respectively by more than 73.3%, 10%, and 7.2% compared with unfilled VE. It is well known that shifting degradation temperatures alters the thermal stability of composites^51^. In general, the activation reaction has improved the thermal behavior of VE-GF composites. 25% VE-AGF is the most thermally stable composite.

**Table 1:** Primary degradation temperatures of VE-FG and VE-AFG composites.

Degradation	VE	20% VE-FG	20% VE-AFG	25% VE-FG	25% VE-AFG
T 0.1 (°C)	210	314	342	356	364
T 0.25 (°C)	349	373	375	377	384
T 0.5 (°C)	375	394	396	397	402
Res %	2.6	13.6	13.6	17.2	17.3

### Mechanical properties

**Figure 3:**
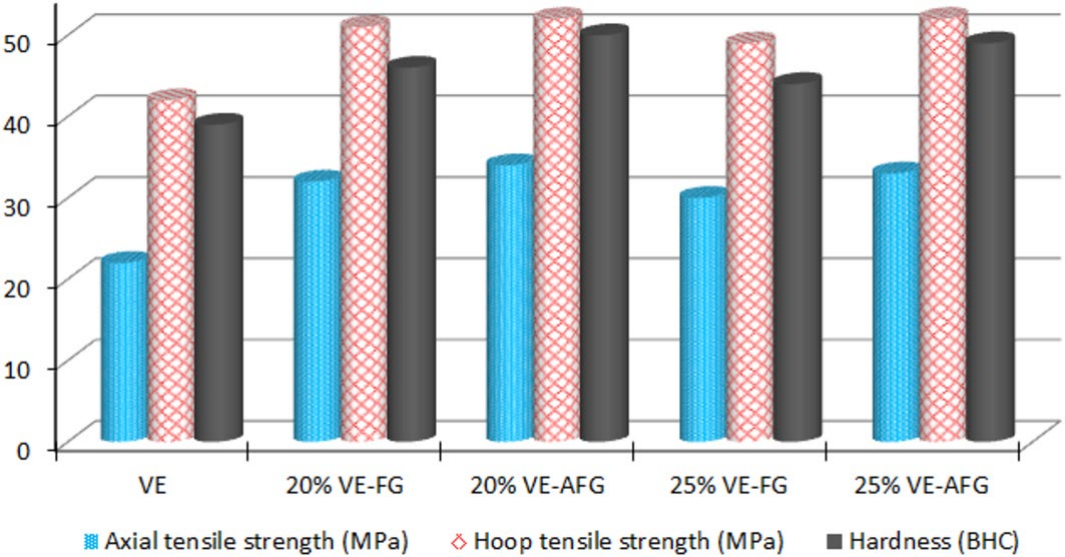
Mechanical properties of VE-FG and VE-AFG composites.

The effect of silane activation on the mechanical properties of proposed composites, compared with blank polymer, is illustrated in Fig. 3. Strengths in axial and hoop directions and hardness values are tested. It is clear that activation of fiber glass filler has promoted all mechanical characteristics, even with the 25% composite, which has lower strengths and hardness than the 20% concentration. For the 20% concentration, the activation reaction has increased the axial strength, hoop strength, and hardness by 6.3%, 2%, and 8.7%, respectively. However, the same values were increased by 10%, 6.1%, and 11.4% for the 25% composite. The related enhancement in mechanical properties appears distinctly in the 25% VE-AFG, due to the successful activation reaction by silane and the efficiency of filling technique. The treatment reaction was found to be essential for the enhancement of the mechanical behavior of FRP, even in the case of some laminated metallic layers^52^. The enhancement in mechanical properties was noticed previously after modification and functionalization in several ways^53,54^. It is proposed that silane material in activated filler can reinforce the interface between particles of fiber glass and the vinylester matrix, resulting in improved hardness and strength in composites. Compared to the literature, the related mechanical characteristics were promoted by treatment with “glycidoxypropyltrimethoxysilane”, in addition to the curing process and crosslink density at a concentration of 2.5%. Also, silane-modified fiber glass was used for reinforcing polysulfone with high content; the mechanical and physical characteristics were altered by means of modification due to the strong interface^55,56^. With another type, the aminopropyltriethoxysilane has increased the strength and impact of the composites^57^. Here, the silane activation improved the mentioned values in both hoop-cut and axial-cut specimens. Basically, the pipe’s strengths achieved in hoop and axial specimens, respectively, refer to the effective fiber glass distributed in the hoop and axial directions during continues winding processing. Generally, the activation of FG led to an enhancement of all mechanical characteristics; this positive effect is also achieved for the composition, which has lower mechanical performance.

### Biological stability

**Figure 4:**
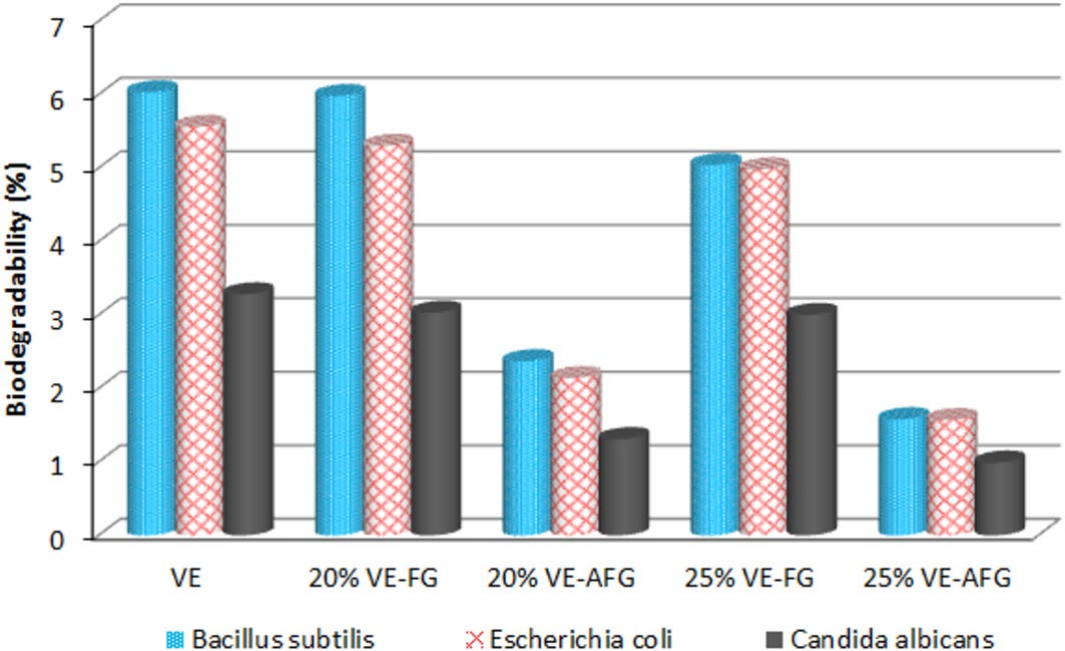
Biological stability of VE-FG and VE-AFG composites in different microorganisms.

This section discusses the stability of proposed composites against biological attack. Vinylester and similar thermosetting polymers, such as polyester and epoxy, are considered to be hardly affected by the different Gram microbial species. Moreover, it was reported that silane-based compounds show stability against decay affected by different types of microorganisms and bacteria; the related composites are physically, mechanically, and surface-improved^58–60^. Figure 4 indicates the biodegradability % of untreated and silane-activated composites in the selected strains, compared with blank vinylester. As noticed for blank and untreated composites, the biodegradation percentage is approximately low; it almost records values similar to the 20% VE-GF composites. However, with the activation reaction, the biodegradability shows sharply lower percentages. This is noticed in both 20% and 25% VE-AGF composites, especially the later specimen, where it decreased by about 74%, 72%, and 70%, compared with blank vinylester, after growing *Bacillus subtilis*, *Escherichia coli*, and *Candida albicans* microorganisms, respectively. The resistance to biodegradability may be due to the hydrophobicity gained occurred after modification by silane activator. Such results generally indicate that the prepared activated composites can resist attacking grown microorganisms after the proposed attachment between all reactants as presented in Scheme 2. This prevents surface deterioration, leading to enhanced surface and mechanical stability.

### Water absorptivity

**Figure 5:**
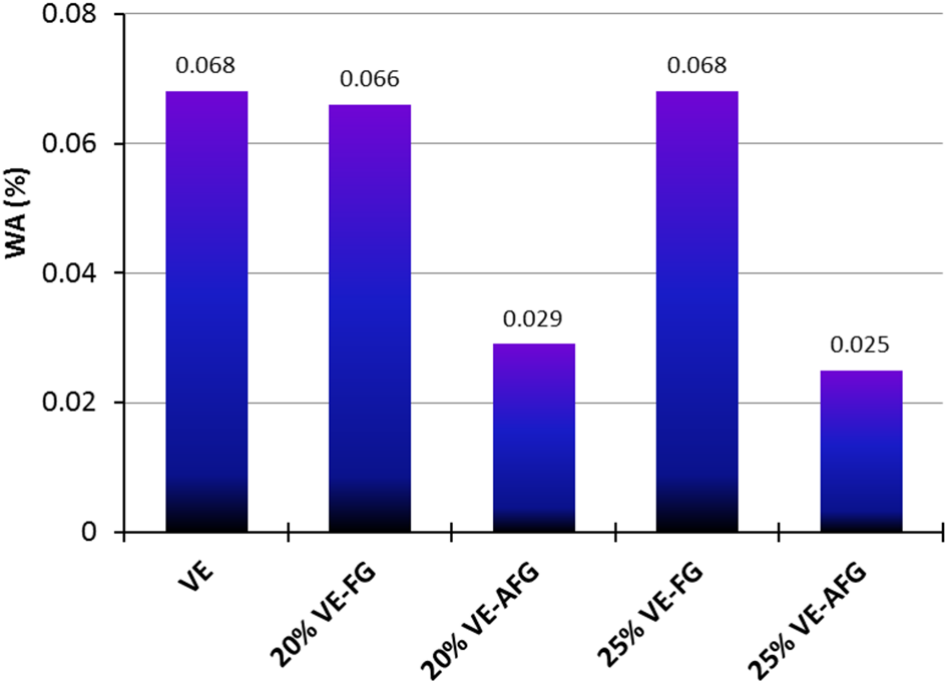
Water absorptivity of VE-FG and VE-AFG composites.

The effect of silane activation on the water absorptivity (WA) of the selected composites is studied, as seen in Fig. 5. The recorded percentages show that 20% and 25% VE-FG composites nearly have similar WA% under the same testing conditions. However, the 20% and 25% VE-AFG composites recorded lower percentages of 0.029% and 0.025%, respectively. The lowest percentage is recoded by the 25% VE-AFG composite, minimizing WA% by more than 62%. This distinguished value reflects the more stable 25% VE-AFG composite after absorbing the minimum content of water, compared with other composites and blank VE. The activation reaction of fiber glass has promoted the interfacial interaction at the vinylester-fiber glass interface; consequently, the ability to prevent moisture is increased. Basically, the water absorptivity of FRP strongly affects the properties of the composites and hybridizations^61^. Similar behavior was reported for diffident composite types regarding less absorbed moisture after progressive surface treatments for the filling materials taken^62,63^. It can be concluded that the activation of fiber glass has increased the stability of vinylester polymer against water absorptivity. Such behavior is a good physical property for the proposed vinyl ester-fiber glass pipe system.

## Conclusion

- A high-performance composite, recommended for water pipe systems, was accomplished by continuous winding processing based on different concentrations of vinylsilane-activated fiber glass single filler and vinylester matrix.
- The crosslinking occurred at unsaturation sites in activated fiber glass and vinylester. The activation reaction facilitated the interaction between inorganic fiber glass and organic matrix with greater compatibility.
- The activation reaction improved the thermal stability, especially with the 25% composite, due to strengthening the fiber glass-vinylester interface, and deformation at higher temperatures.
- Vinylsilane-activated fiber glass promoted the mechanical properties, even with 25% composite, which has lower strengths. The most improved 20% concentration has increased axial strength, hoop strength, and hardness by 6.3%, 2%, and 8.7%, respectively.
- The prepared activated composites resisted attacks by different microorganisms, where stability to biodegradation was induced by the silane activation reaction. The biodegradability showed sharply lower percentages with all composites, especially the 25% VE-AGF one, due to the hydrophobicity gained after modification of fiber glass.
- The resistance against water absorption was increased after activation. The proposed composites absorbed lower percentages of water, particularly with the 25% VE-AFG composite, which was reduced by more than 62%, reflecting a more stable concentration.
- The given analysis data lets the proposed activated vinylester-fiber glass be applied to pipe systems with enhanced physical and mechanical characteristics, in addition to its stability toward microorganisms attacking.

## Data Availability

The datasets used and/or analysed during the current study available from the corresponding author on reasonable request.
